# Toward a Broader View of Ube3a in a Mouse Model of Angelman Syndrome: Expression in Brain, Spinal Cord, Sciatic Nerve and Glial Cells

**DOI:** 10.1371/journal.pone.0124649

**Published:** 2015-04-20

**Authors:** Mark D. Grier, Robert P. Carson, Andre Hollis Lagrange

**Affiliations:** 1 Department of Neurology, Vanderbilt University Medical Center, Nashville, TN 37232–8552, United States of America; 2 Department of Pediatrics, Vanderbilt University Medical Center, Nashville TN 37232–8552, United States of America; 3 Department of Neurology, Tennessee Valley Veterans Administration, Nashville, TN 37212, United States of America; Western University of Health Sciences, UNITED STATES

## Abstract

Angelman Syndrome (AS) is a devastating neurodevelopmental disorder characterized by developmental delay, speech impairment, movement disorder, sleep disorders and refractory epilepsy. AS is caused by loss of the Ube3a protein encoded for by the imprinted *Ube3a* gene. *Ube3a* is expressed nearly exclusively from the maternal chromosome in mature neurons. While imprinting in neurons of the brain has been well described, the imprinting and expression of Ube3a in other neural tissues remains relatively unexplored. Moreover, given the overwhelming deficits in brain function in AS patients, the possibility of disrupted Ube3a expression in the infratentorial nervous system and its consequent disability have been largely ignored. We evaluated the imprinting status of *Ube3a* in the spinal cord and sciatic nerve and show that it is also imprinted in these neural tissues. Furthermore, a growing body of clinical and radiological evidence has suggested that myelin dysfunction may contribute to morbidity in many neurodevelopmental syndromes. However, findings regarding Ube3a expression in non-neuronal cells of the brain have varied. Utilizing enriched primary cultures of oligodendrocytes and astrocytes, we show that *Ube3a* is expressed, but not imprinted in these cell types. Unlike many other neurodevelopmental disorders, AS symptoms do not become apparent until roughly 6 to 12 months of age. To determine the temporal expression pattern and silencing, we analyzed Ube3a expression in AS mice at several time points. We confirm relaxed imprinting of *Ube3a* in neurons of the postnatal developing cortex, but not in structures in which neurogenesis and migration are more complete. This furthers the hypothesis that the apparently normal window of development in AS patients is supported by an incompletely silenced paternal allele in developing neurons, resulting in a relative preservation of Ube3a expression during this crucial epoch of early development.

## Introduction

Angelman syndrome (AS) is a neurodevelopmental disease characterized by developmental delay, speech impairment, movement disorders, sleep disorders and refractory epilepsy[1]. These symptoms have a devastating impact on the quality of life for individuals with AS and their caregivers. AS results from the loss of neuronal Ube3a protein, an E3 ubiquitin ligase derived from the *Ube3a* gene[2]. *Ube3a* is maternally imprinted in the brain, such that it is expressed nearly exclusively from the maternal chromosome while the paternal chromosome is epigenetically silenced[3,4]. *Ube3a* imprinting is regulated by a neuron-specific anti-sense *Ube3a* transcript that prevents transcription of *Ube3a* from the paternal chromosome[5,6]. Unlike some other neurodevelopmental disorders, infants with AS develop normally for 6 to 12 months before developmental delay becomes evident[7], Furthermore, other than relative microcephaly seen in some patients, gross brain morphology of AS patients is generally normal, with only subtle structural abnormalities reported. Together, this supports the hypothesis that residual paternal Ube3a may be present in early brain development before the paternal allele is silenced. Recently published work in the visual cortex indicates that incomplete imprinting of *Ube3a* results in approximately 30% of wild type (WT) protein level is present at P5[8] in *Ube3a* maternally deficient (m-/p+) mice. This suggests that there is an early period of preserved Ube3a expression in (m-/p+) mice, from an unsilenced paternal allele, might serve to protect normal neurodevelopment and cellular function.

To date, Ube3a expression in neural tissue outside of the brain has been largely unexplored. However, work with the drosophila homolog of *Ube3a*, *dUbe3a*, has shown that loss of this protein results in slowed dendritic growth and reduced dendritic branching in peripheral nerves[9]. Additionally, somatosensory-evoked potentials in a subset of AS patients are abnormal[10]. This suggests that if *Ube3a* is imprinted in peripheral or spinal nerves, this would provide a reason to evaluate the consequences of loss of Ube3a in neurons outside of the brain.

Much of the work presented here builds upon previously published work showing that *Ube3a* imprinting is relaxed in early postnatal cortex and *Ube3a* is not imprinted in astrocytes or oligodendrocyte[11]. However, there have been conflicting reports on the expression of Ube3a in astrocytes and glial cells and the timing of *Ube3a* silencing in development[4,8,12,13]. In order to unify the conflicting results of previous work, we chose pursue a more extensive evaluation of Ube3a temporal expression and imprinting in a variety of nervous tissues and cell types which revealed that *Ube3a* imprinting in neurons is more widespread than previously appreciated, while non-neuronal cells express *Ube3a* biallelically.

## Methods

### Mouse Breeding

*Ube3a* deletion mice, derived by Jiang were used for all studies[14]. Mice were maintained on C57BL/6J background. WT males (*Ube3a* m+/p+) were crossed with AS females (*Ube3a* m−/p+) to produce WT and AS littermates. PCR analysis was performed on tail tissue (harvested prior to P10) or ear punches (harvested after P10) to determine genotypes. PCR primers used as followed: P1 (Common), 5′-CCAATGACTCATGATTGTCCTG-3′; P2 (WT reverse), 5′-TCAAACATTCCAAGTTCTCCC-3′; and P3 (mutant), 5′-TGCATCGCATTGTGTGAGTAGGTGTC-3′. PCR protocol: 94°C for 3 minutes, (94°C for 30 seconds, 58.3°C for 1 minute, 72°C for 1 minute) x 30 cycles. We utilized a modified protocol as the previously published PCR primers have errors in the primer sequences compared to the curated sequence[14]. The protocol supplied by The Jackson Laboratory using these primers has an annealing temperature that results in non-specific amplification. Male and female mice were used in all experiments.

### Ethics

This study was carried out in strict accordance with recommendations in the Guide for the Care and use of Laboratory Animals of the National Institute of Health. All procedures were approved by the Vanderbilt Institutional Animal Care and Use Committee (IACUC) under protocol number M/11/059.

### Tissue Collection

Brain tissue was collected from mice aged 0 to 42 days. Mice were decapitated and the brains were removed quickly, chilled in ice cold artificial cerebrospinal fluid (aCSF) containing NaCl (125 mM), KCl (2.5 mM), Na_2_HPO_4_ (1.25 mM), MgCl_2_ (1.3 mM), CaCl_2_ (2.0 mM), glucose (10 mM), and NaHCO_3_ (25 mM) and dissected to isolate cortex, subcortex and cerebellum. All tissue was flash frozen in liquid nitrogen and stored at -80°C until being prepared for Western blot.

### Western Blot

Tissue was homogenized with a sonicator (QSonica) in a modified RIPA buffer containing (50 mM Tris (pH = 7.4), 150 mM NaCl, 1% NP-40, 0.2% sodium deoxycholate, 1 mM EDTA) with protease inhibitors (Sigma). Protein concentration was determined by a Bradford assay (Bio-Rad), and samples were diluted to a final concentration of 1 to 3 μg/μL in loading buffer containing β-mercaptoethanol. Samples were denatured at 60° C for 10 minutes before being loaded onto a 10% SDS gel (Bio-Rad). Proteins were transferred in a Tris-glycine transfer buffer onto Immobilon-FL PVDF membranes (Millipore). Membranes were then blocked in 4% milk overnight at 4° C. Membranes were incubated with primary antibody in PBS-T with 0.1% Tween-20 (PBS-T) for 2 hours at room temperature. Primary antibodies used: Ube3a 1:2000 (Sigma E6855, mouse monoclonal), Ube3a 1:1000 (Cell Signaling D10D3, rabbit monoclonal), GFAP 1:1000 (Cell Signaling 3670, mouse monoclonal) and actin 1:50000 (Millipore MAB1501, mouse monoclonal). Membranes were washed at least three times for 10 minutes in PBS-T and then incubated with fluorescent secondary antibodies at 1:10000 (LI-COR, IRDye800CW goat anti-mouse, IRDye680RD goat anti-rabbit) in PBS-T for 1 hour at room temperature. Membranes were then washed three times for at least 10 minutes with PBS-T. Imaging was performed on a LI-COR Odyssey fluorescence scanner and data was analyzed with Odyssey imaging software.

### Antibody Validation

To better evaluate Ube3a protein expression we first characterized a newly available antibody against Ube3a (Cell Signaling) and compared it to the best characterized antibody against Ube3a (Sigma Anti-Ube3a Clone 330). Each antibody was run against a panel of tissues including lysates from brain, spinal cord and sciatic nerve as well as several non-neurally derived tissues including liver, heart, lung and kidney. When comparing the banding pattern produced in WT lysates to *Ube3a* null littermates, we found that the Sigma Ube3a antibody is highly selective, recognizing no non-specific bands in the 100kD range in any tissue. In contrast, the Cell Signaling antibody recognized non-specific bands in the same molecular weight range as Ube3a in cortical, cardiac and spinal cord lysates. However, no non-specific bands were detected in the sciatic nerve or liver, kidney or lung (S1 Fig). We next compared both antibodies in cortical lysates from P42 mice to determine suitability for use in our studies. When run against lysates from WT, AS (*Ube3a* m-/p+), paternal deletion (*Ube3a* m+/p-) and *Ube3a* null mice we found the expected pattern of paternal deletion being indistinguishable from WT, a small residual protein content in AS mice and tissue from *Ube3a* null mice having no protein detected in the 100kD range with either antibody. However, there was a non-specific band that migrates at a slightly higher molecular weight than Ube3a detected by the Cell Signaling antibody (S1 Fig). Therefore the Sigma antibody should be preferred for quantitative Western blot, however the Cell Signaling antibody was sufficient to provide protein verification of genotypes with the flexibility of a rabbit primary antibody.

### Primary culture

Primary astrocyte and oligodendrocyte cultures were derived with modification of a shaking protocol described previously[15]. Following sacrifice by decapitation, P0-P2 mouse brains were placed in ice-cold MEM and cortex was isolated from ventral structures and meninges. Cortex was triturated and dissociated with papain for 20 minutes at 37° C. Following incubation, papain was inactivated by addition of mixed glial culture media (MGCM; DMEM with 10% FBS, 1x pen-strep), and tissue triturated with a flame-polished Pasteur pipette and plated in poly-L-lysine (PLL) coated T25 flasks and incubated at 37° C and 8.5% CO_2_. Two-thirds media changes were done on DIV3 and DIV6, with 5 μg/mL insulin supplemented at DIV6. Following a pre-shake at 50 RPM for 45 minutes to remove microglia, on DIV9, oligodendrocyte precursors were isolated by shaking overnight at 220 RPM to make both enriched oligodendrocyte and astrocyte cultures. Following shaking, suspended oligodendrocyte precursors were plated into PLL coated 8-well chamber slides at a density of 20,000 cells/well in OL media (DMEM supplemented with BSA, progesterone, putrescine, sodium selinite, 3,3’,5-triiodo-L-thyronine, insulin, glutamine, holo-transferrin, B27, and FBS) and cultured for 7–9 days at 37° C and 8.5% CO_2_. Astrocytes remaining in the T25 flask were washed with PBS and dissociated with 0.25% trypsin in Hank’s buffered saline solutions (HBSS). Dissociated cells were plated in PLL coated 24-well plates at a density of 30,000 cells per well for IF or into 6-well culture dishes for Western blot. Cells were grown for 2–3 days at 37° C in 5% CO_2_ before being fixed or harvested for Western blot.

### Immunofluorescence

Cultured astrocytes and oligodendrocytes were fixed with 4% PFA for 30 minutes and washed 2x with PBS before being blocked with 5% goat serum and 0.1% Triton X-100. Primary antibodies (Sigma Ube3a 1:300, MBP: 1:200 and GFAP 1:500) were diluted in blocking buffer and samples were incubated overnight. Secondary antibodies were diluted in blocking buffer (Goat Anti-Rat Alexa-488 (Life Technologies), Goat Anti-Rabbit Alexa-488 (Life Technologies) and Goat Anti-Mouse 680LT (Li-Cor), all 1:500) and incubated for one hour at room temperature. Cells were washed twice with PBS and coverslipped with Vectashield with DAPI prior to imaging. All imaging was performed on an Evos-FL microscope.

### Statistics

All statistical analyses were performed using GraphPad Prism (version 5.03, La Jolla CA) software. Unless otherwise specified, an unpaired t-test was used to compare two age-matched groups. A one-way ANOVA with Tukey’s post hoc test was used to compare the residual paternal Ube3a expression in AS mice at different ages.

## Results

### *Ube3a* is imprinted at multiple levels within the neuraxis

**Fig 1:**
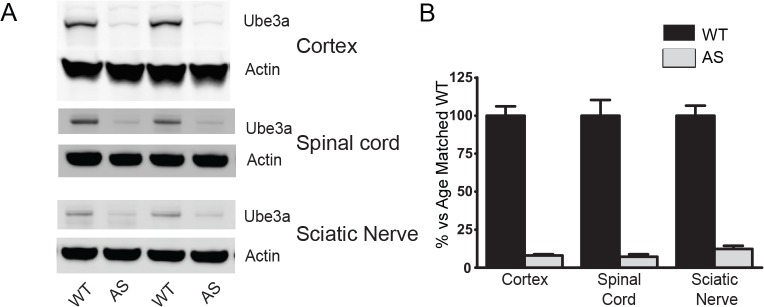
*Ube3a* is imprinted in brain, spinal cord and sciatic nerve. A) Representative data from cortex, spinal cord and sciatic nerve lysates. B) Lysates from P42 spinal cord and sciatic nerve from AS animals have a reduction of Ube3a expression similar to that seen in cortex. n = 13–19 per group.

Clinical and basic research to date has focused nearly exclusively on disrupted Ube3a in the brain and imprinting in neurons[4]. This is likely due to the fact that the overwhelming morbidity faced by AS patients are related to CNS dysfunction. Moreover, given the presence of peripheral motor reflexes and lack of a clear spinal phenotype, it might easily be assumed that spine and peripheral nerve function are relatively spared AS, although this has not been studied in detail. To determine the imprinting status of *Ube3a* throughout the neuraxis, we first confirmed the imprinting of *Ube3a* in P42 murine cortical lysates. We next determined that Ube3a is highly expressed in the spinal cord and sciatic nerve in WT mice and it is significantly reduced in AS mice. As expected, paternal Ube3a is expressed at very low levels, approximately 5–10% of WT in AS mice in all neural tissues assayed (Fig 1A and 1B). Ube3a is not expressed in *Ube3a* knockout (m-/p-, KO) tissue (S1 Fig). These data confirm that *Ube3a* is imprinted in both the spinal cord and in peripheral nervous tissue.

### *Ube3a* is expressed, but not imprinted in primary astrocyte and oligodendrocyte cultures from AS mice

**Fig 2:**
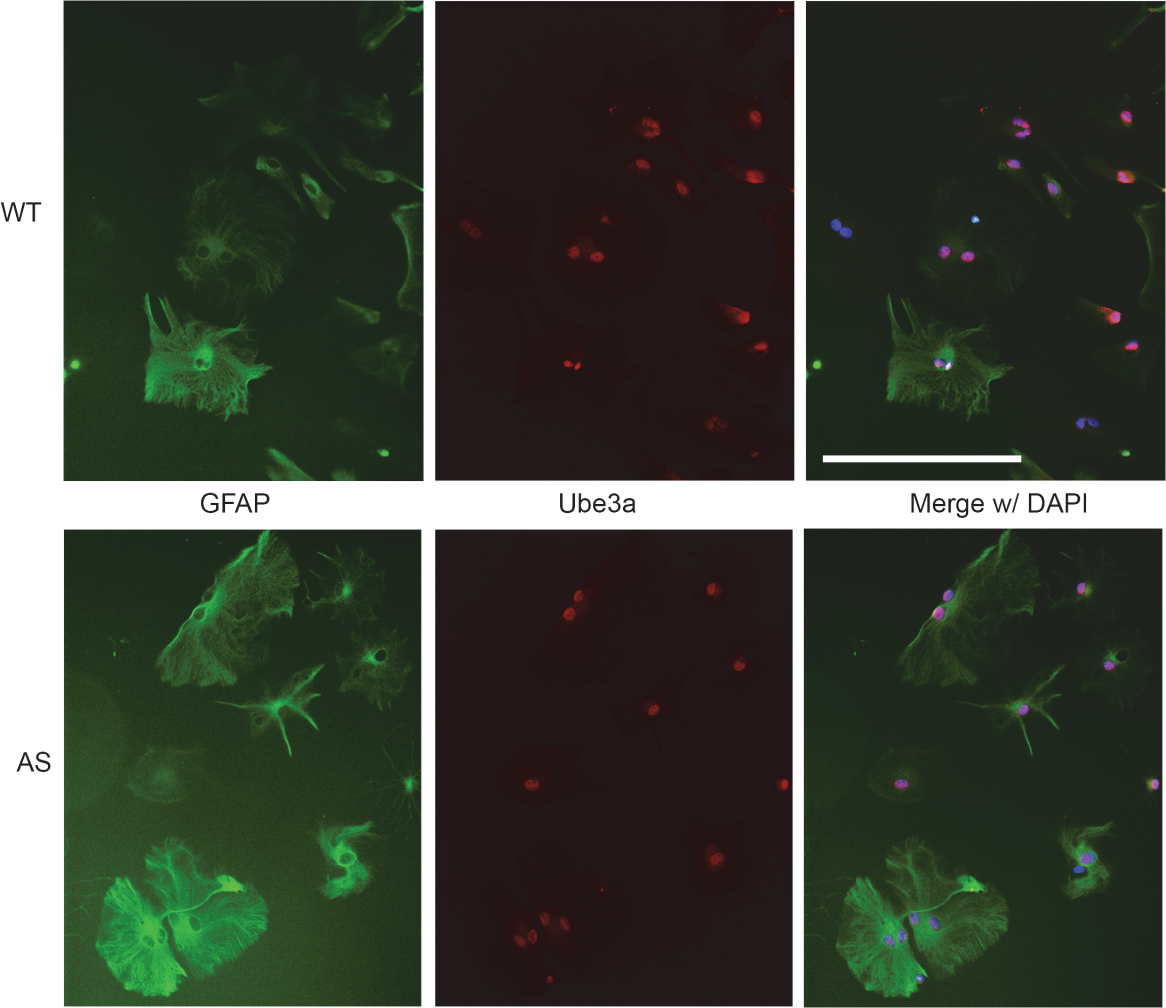
Ube3a is expressed, but not imprinted in cultured astrocytes from AS animals. A) WT upper panels, AS lower panels. Left to right: GFAP (a marker for astrocytes), Ube3a and merge of GFAP with Ube3a and DAPI. Ube3 expression is most apparent in the nucleus of GFAP positive cells, with lower levels of expression throughout the cytosol. DAPI colocalizes with nuclear Ube3a. Scale bar represents 200 μm.

**Fig 3:**
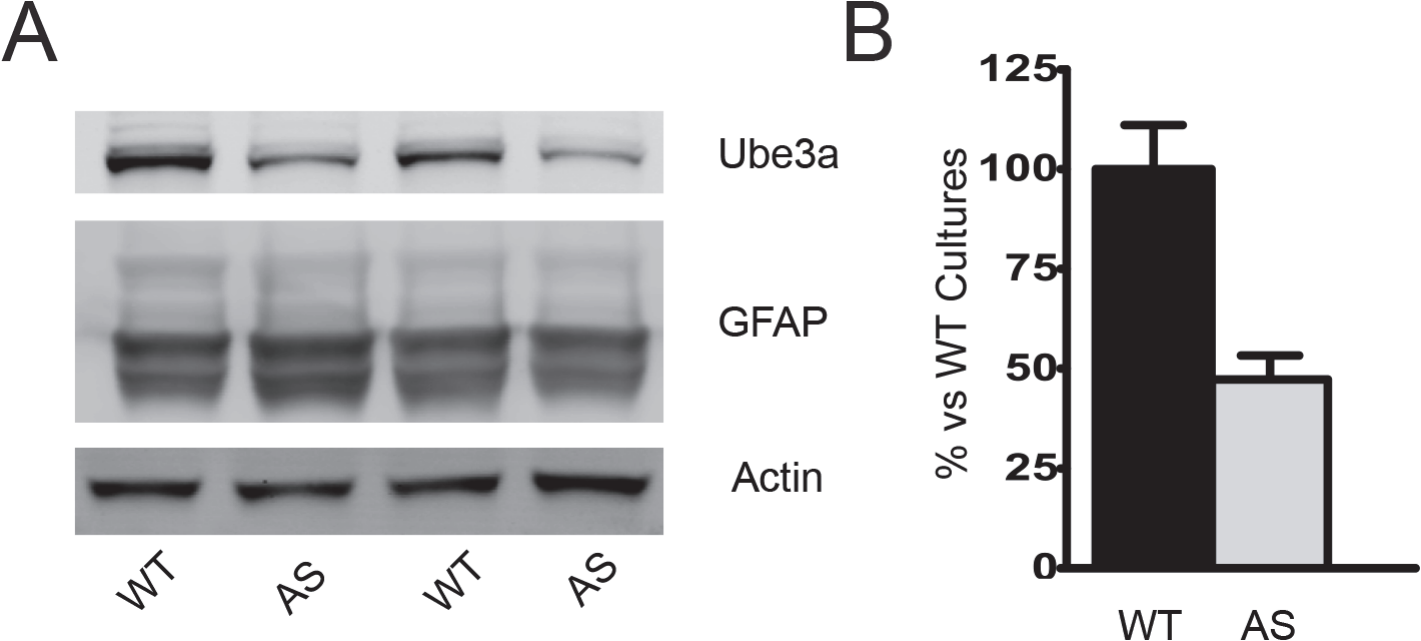
Ube3a is not imprinted in cultured astrocytes. A) Representative data from enriched astrocyte cultures showing expression of Ube3a and high expression of GFAP, confirming astrocyte enrichment. B) Quantification of data showing an approximately 50% reduction in Ube3a in cultured astrocytes from AS animals. This is not different from 50% as determined by a one sample t-test against 50%, the expected reduction for a biallelically expressed protein. n = 6–7 independent cultures from individual animals per group.

A growing body of clinical evidence has suggested that myelin dysfunction may be a contributing factor to morbidity in a variety of neurodevelopmental syndromes[16–18]. Recent diffusion tensor imaging (DTI) studies have found altered white matter tracts, thinned corpus callosa and delayed myelination and in Angelman patients[19–21]. While earlier studies suggest that *Ube3a* imprinting occurs primarily in neurons, recent evidence suggests *Ube3a* is not imprinted in glial cells [12,22]. In addition, recent work from our lab has revealed disrupted myelin protein expression in AS mice, thus we sought to determine the extent to which Ube3a is expressed in glial cells and whether the expression is maternally imprinted. Expression of Ube3a was determined in primary astrocyte and oligodendrocyte cultures derived from WT and AS mouse cortex. Immunofluorescent micrographs of glial fibrillary acid protein (GFAP) positive astrocytes from WT mice demonstrated intense nuclear and diffuse cytosolic staining for Ube3a (Fig 2, upper panels). While the cultures were highly enriched, Ube3a immunoreactivity could occasionally also be noted in GFAP-negative cells. Astrocytes derived from AS mice showed a reduction in Ube3a immunoreactivity compared to WT astrocytes, but not the complete loss that would be expected were it imprinted (Fig 2, lower panels). To better quantitate the difference in expression, protein lysates from the enriched astrocyte cultures were analyzed by Western blot and show a reduction of Ube3a of approximately 50% compared to WT control (Fig 3). This is consistent with biallelic expression and supports that *Ube3a* is not imprinted in astrocytes.

**Fig 4:**
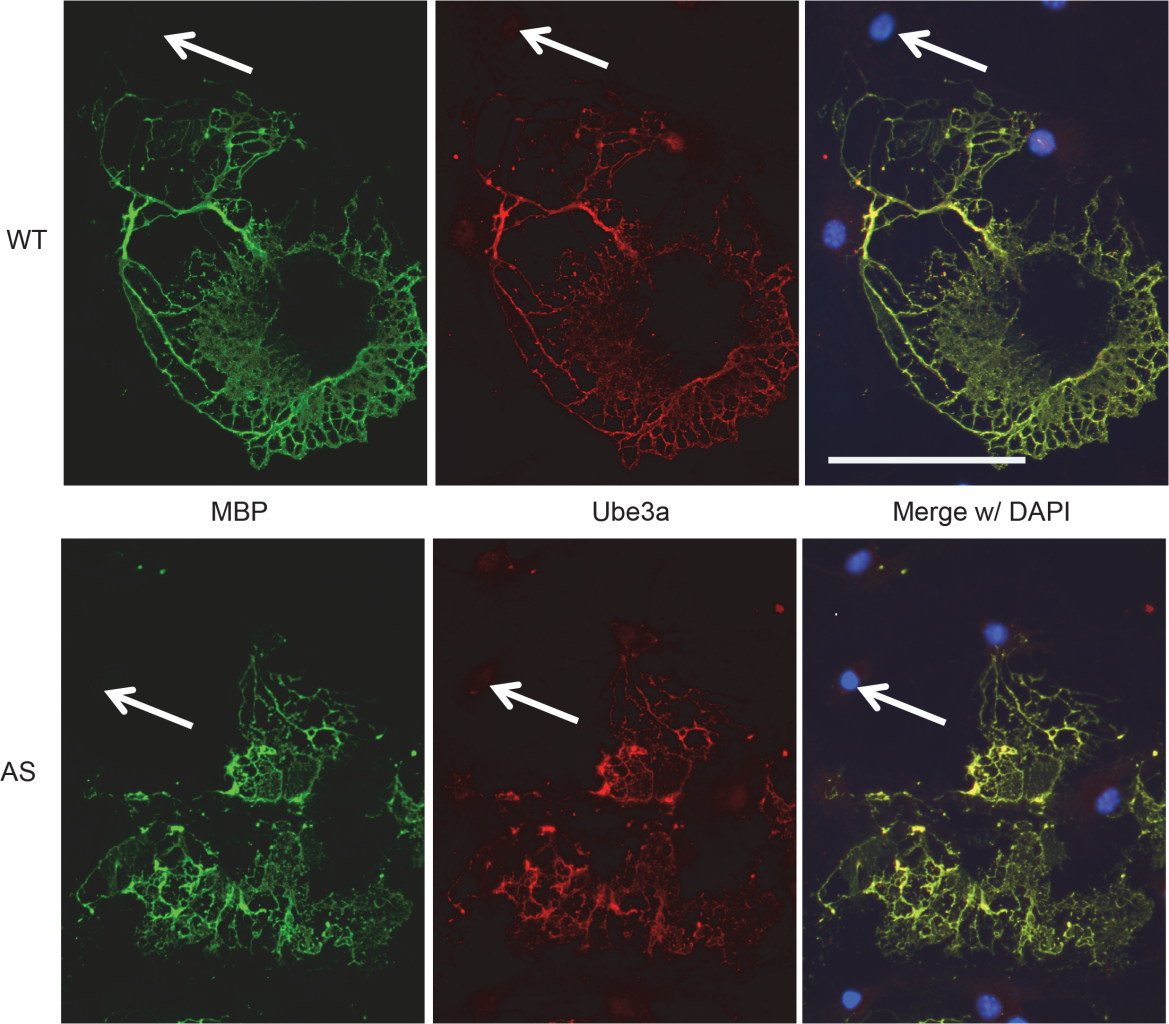
Ube3a is expressed, but not apparently imprinted in cultured oligodendrocytes from AS animals. WT upper panels, AS, lower panels. Left to Right: Myelin Basic Protein (MBP, a marker for oligodendrocytes), Ube3a and merge with DAPI. Ube3a is expressed throughout oligodendrocytes as shown by robust colocalization with MBP in both WT and AS oligodendrocytes. Arrows highlight Ube3a (+), MBP (-) cells, likely to be contaminating astrocytes. Scale bar represents 100 μm.

Likewise, to determine if *Ube3a* is imprinted in oligodendrocytes, expression of Ube3a was evaluated in primary cultures of mature oligodendrocytes. Myelin basic protein (MBP)-positive mature oligodendrocytes from WT mice show robust expression of Ube3a in the nucleus as well as high levels of expression in the cytosol, in contrast to astrocytes which demonstrated a somewhat reduced immunoreactivity in the cytosol compared to the nucleus (Fig 4, upper panels). The pattern of paternal Ube3a expression in AS oligodendrocytes was not significantly different from that of the WT animals (Fig 4, lower panels). The high level of expression of Ube3a in AS oligodendrocytes strongly suggests that *Ube3a* is not imprinted in oligodendrocytes. Unfortunately, given the low yield of oligodendrocytes obtained in culture, we were unable to formally quantify Ube3a protein expression by Western blot.

### The paternal allele of Ube3a is silenced at birth in most brain regions, but not in the cortex

**Fig 5:**
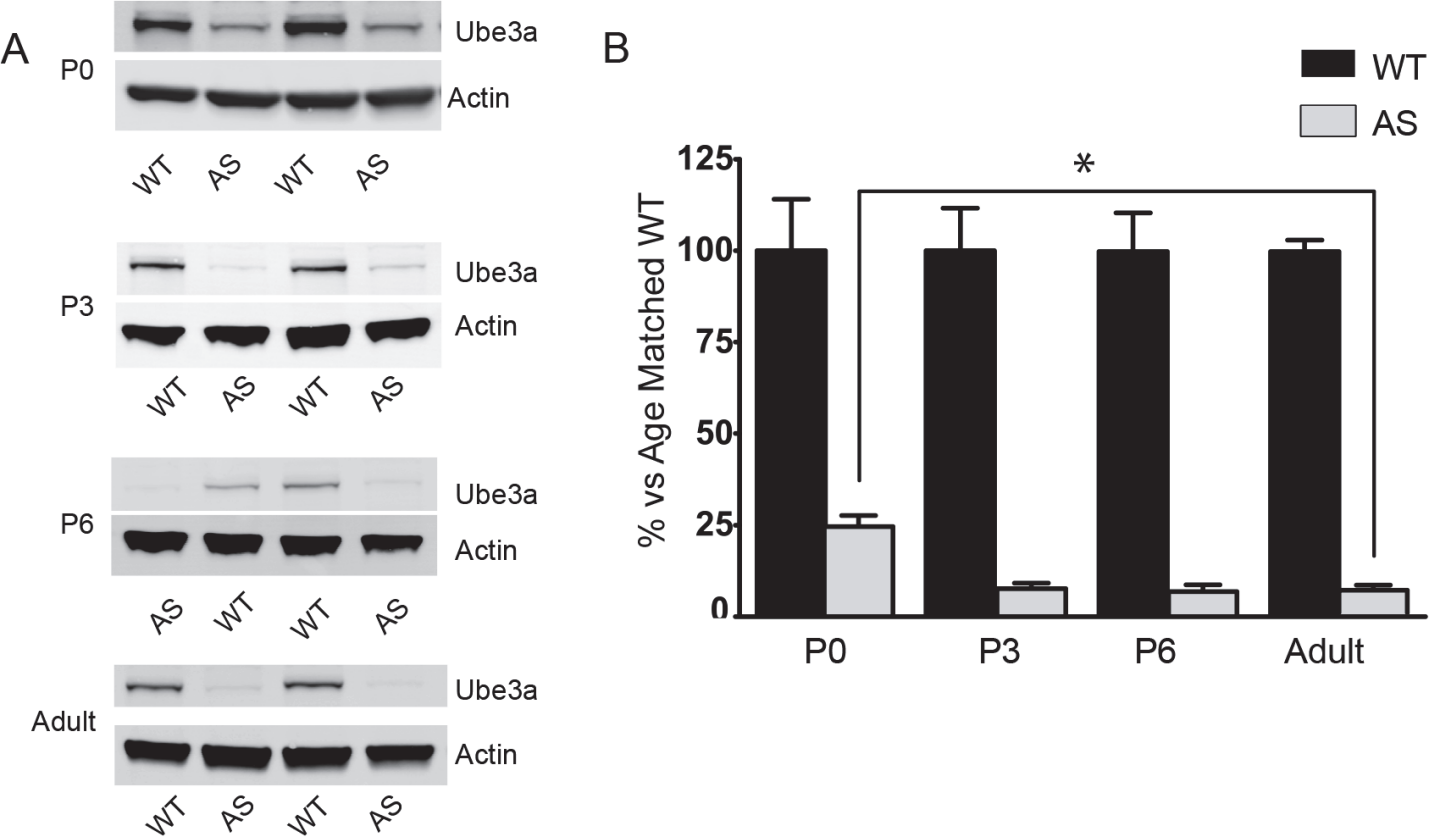
Ube3a expression time course in cortical lysates. A). Representative data for expression of Ube3a in P0, P3, P6 and P42 cortical lysates. B) Quantification of Ube3a expression at various time points. P0 cortical lysates express approximately 25% of WT protein, compared to approximately 5% of WT expression at P3 and later. *** indicates P ≤.0001 by a one-way ANOVA with Tukey's multiple comparison test comparing AS animals at each time point. n = 7–10 per group.

**Fig 6:**
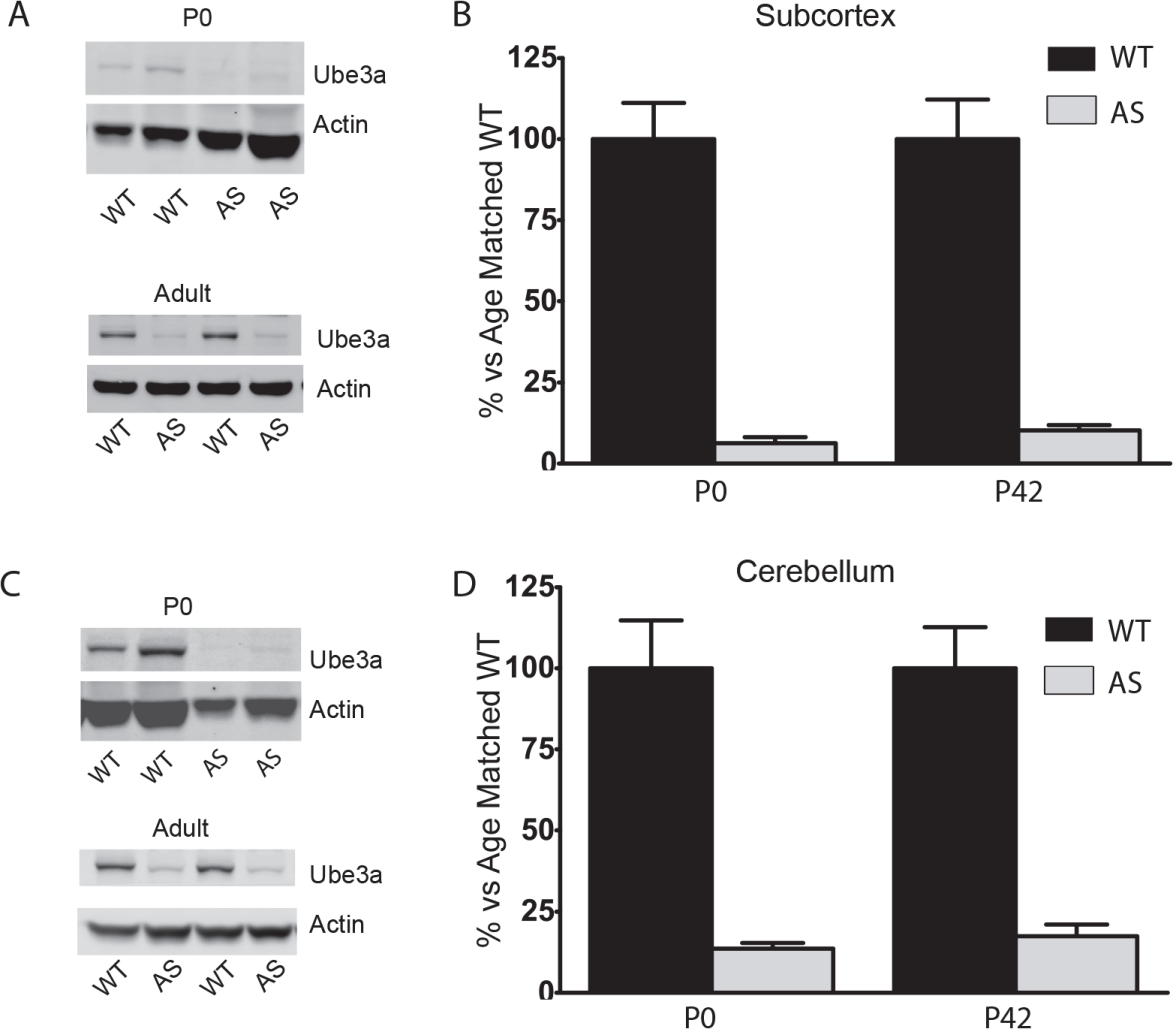
Ube3a expression in other brain regions A) Representative data for Ube3a expression in P0 and P42 subcortical lysates (thalamus and hypothalamus). B) Quantification shows approximately 5–10% residual paternal Ube3a at birth and P42. c) Representative data for Ube3a expression in cerebellar lysates. d) Quantification shows 10–15% residual paternal Ube3a at P0 and P42. N = 3–5 per group.

As much of the research done on AS mice has been carried out in adult animals, the developmental profile of *Ube3a* expression and consequences of lingering paternal Ube3a expression in early development had been largely ignored until recently. A recently published report indicates that imprinting of *Ube3a* is incomplete in neonatal AS mice. This is believed to be due to incompletely silenced paternal allele[8]. Additionally, work in induced pluripotent stem cells (iPSCs) derived from AS patients suggests that the Ube3a-ATS transcript responsible for silencing the paternal allele of *Ube3a* is not expressed until very late in neurogenesis[23]. To determine the developmental expression pattern of Ube3a in mice, cortical lysates from several developmental time points were assayed for Ube3a expression. As expected, P42 mice had very little, <10% of WT, Ube3a protein in the cortex. However, at P0 we observed approximately 25% of WT Ube3a expressed in cortical lysates (Fig 5). This level of expression rapidly dropped over the next 3 days and was indistinguishable from P42 levels by P3 (Fig 5). This is in contrast to sub-cortical and cerebellar tissue in which Ube3a expression was at near-P42 levels at birth (Fig 6). These data further support a regional and developmental regulation for imprinting of *Ube3a* in neurons.

## Discussion

Like many neurodevelopmental disorders, much of the morbidity in AS is attributed to brain neuronal dysfunction. As such, research to date has focused on consequences of maternal Ube3a deficiency in cortical, hippocampal and cerebellar neurons. With advanced MRI based techniques, we are gaining a greater appreciation of white matter abnormalities in neurodevelopmental disorders, including AS[24–26]. While, the imprinting status of *Ube3a* has been well described in the brain of AS mice, fewer studies have focused on the timing of imprinting, and the imprinting status in glia, spinal cord and peripheral nerves. Here we show that expression of Ube3a is markedly reduced in the spinal cord and sciatic nerve, consistent with imprinting of *Ube3a* in these tissues. We also show that *Ube3a* is not imprinted in glia and that residual paternal Ube3a is expressed in neurons early in development.

*Ube3a* imprinting in the spinal cord has been demonstrated in the spinal cord of paternal Ube3a-YFP expressing mice. Administration of topotecan, a treatment known to unsilence the paternal allele of *Ube3a* in the cortex of AS model mice[27], also significantly increases the expression of paternally derived YFP labeled Ube3a in neurons of the spinal cord. Here we demonstrate that native paternal *Ube3a* is also imprinted, indicating that the YFP tag has no impact on the silencing of the gene. Imprinting of *Ube3a* in the spinal cord and peripheral nerves suggest that some characteristics of Angelman Syndrome such as hypo-responsiveness to tactile stimuli and sensory processing difficulties [28] may be due in part to alterations in the peripheral nervous system rather than strictly deficits in the brain. Further, nerve conduction and evoked potential abnormalities in peripheral tissues, should be evaluated as a more accessible biomarker for AS.

When considering the role of Ube3a in neuronal function there are several findings that raise additional questions. One such finding is that adult AS mice express a small amount of paternal Ube3a diffusely throughout the brain [4,12], that may be the result of expression of Ube3a in astrocytes or oligodendrocytes[12]. The non-imprinted expression of *Ube3a* in glial cells has been demonstrated at the mRNA level in primary cultures[29], but there have been conflicting reports on the expression status of *Ube3a* when studied at the protein level in these cell types, ranging from no expression[4] to non-imprinted expression[30]. While it is clear that loss of maternal Ube3a in neurons is detrimental to normal brain function, astrocytes and oligodendrocytes cells also play crucial roles in maintaining normal synaptic physiology [31,32]. Loss or reduction of Ube3a could have profound effects on not only these cell types, but also overall neuronal health and function. To better show the distribution of Ube3a in these cell types, we utilized enriched primary cultures of astrocytes and oligodendrocytes to provide a detailed study of the expression and imprinting in AS model mice. Enriched cultures of both oligodendrocytes and astrocytes confirmed that paternal *Ube3a* is expressed in both tissues, but it is not imprinted in astrocytes of AS mice. While we were unable to generate enough protein for quantitation of protein from oligodendrocytes, the immunofluorescence data strongly supports that *Ube3a* is not imprinted in oligodendrocytes. Our findings are consistent with a recent study in brain slices demonstrated that Ube3a is expressed in astrocytes and oligodendrocytes from both WT and AS Mice[33]. AS patients demonstrate altered white matter development including delayed myelination and thinned corpus callosum[34–36]. The finding that *Ube3a* is not imprinted in oligodendrocytes suggest that the myelin defects may not be due to altered oligodendrocytes specific deficits, but may instead be the result of altered neuron/oligodendrocyte interactions. Alternatively, it is possible that haploinsufficiency for *Ube3a* may underlie these white matter defects. This hypothesis could be tested utilizing cell type specific *Ube3a* deletions and would provide insight into the role of Ube3a in myelination.

We hypothesized that imprinting of Ube3a may be a late developmental phenomenon. As noted above, this is supported by both prior mouse studies as well as studies in iPSCs [8,23,37]. We confirmed the relaxed imprinting of paternal *Ube3a* in cortical lysates and minimal expression levels in subcortical and cerebellar lysates from neonatal AS model mice. In contrast to previous reports, we observed a rapid silencing of paternal *Ube3a* in the cortex of neonatal mice such that P42 expression levels are reached at or around postnatal day 3. This may be due to the rapid onset of expression of the Ube3a-ATS transcript responsible for silencing the paternal allele of *Ube3a*. Furthermore, there is likely significant variability depending on the cortical region and cell types being studied as suggested by an approximately 30% residual paternal Ube3a expression in P5 visual cortex [8]. The lack of paternal Ube3a expression in P0 subcortical and cerebellar tissue is not surprising as are relatively more mature at birth in mice. This finding is consistent with previous reports showing that only a distinct subset of neural progenitor cells in the hippocampus and cerebellum continue to express maternal Ube3a in adulthood [38], while all other neurally derived cells, save for immature post-mitotic neurons, are largely devoid of Ube3a expression.

These findings extend our knowledge on the expression of *Ube3a* in the nervous system but raise important questions on the role of Ube3a in normal neurogenesis and function. Most significantly, why does a developing neuron tolerate biallelic *Ube3a* expression, while having more than one functional copy in adulthood leads to an autism-like phenotype and reduced excitatory neurotransmission [39]? Evidence has been mounting that copy number variants of the gene segment including *Ube3a* increases autism susceptibility [40,41]. Likewise, a mouse model with increased gene dosage of *Ube3a* demonstrates autism-like behaviors [39]. In addition, AS patients with uniparental disomy, in which two paternally inherited genes are present, generally have a less severe phenotype than deletion patients [42,43], suggesting the incomplete imprinting of *Ube3a* in the brain could be protecting neuronal function to some degree. Moreover, a recent report indicates that at least one behavioral model can differentiate WT, maternal deletion and *Ube3a* null mice in multiple motoric assays and licking behavior. The author presents a model by which residual paternal expression of *Ube3a* protects some behaviors[44]. This could indicate relaxed imprinting of paternal *Ube3a* in early development may be sufficient to support normal cellular function until the silencing mechanism becomes effective. Taken together, this suggests that the level of Ube3a present in the brain lies on a continuum in which too little leads to AS and too much leads to autistic-like phenotype, indicating Ube3a has critical roles in normal neuron function and development.

## Supporting Information

S1 Fig Antibody Optimization. a) Comparison of Ube3a antibodies from Sigma (raised in Mouse) and Cell Signaling (raised in Rabbit) in a panel of tissues known to express Ube3a. WT and KO tissue from tissue was assayed. Top panel shows specificity of Sigma Ube3a with no immunoreactivity at 100 kD in KO tissue in any tissue. Bottom panel shows lack of specificity for Cell Signaling Ube3a with nonspecific bands present at around 100 kD in lysates from cortex, spinal cord, and heart. b) Comparison of antibodies run against WT, paternal deficient Ube3a, AS and KO tissue. As expected, both antibodies have the expected result of WT and paternal deficient sample being nearly indistinguishable, significantly reduced expression in AS tissue and no expression in KO tissue. As noted, a faint non-specific band is recognized by the Cell Signaling antibody.
(TIF)
