# Supplementary figures and images for: Toward a Broader View of Ube3a in a Mouse Model of Angelman Syndrome: Expression in Brain, Spinal Cord, Sciatic Nerve and Glial Cells
Source: PLoS One. 2015 Apr 20;10(4):e0124649. doi: 10.1371/journal.pone.0124649 (PMC4403805; doi:10.1371/journal.pone.0124649)

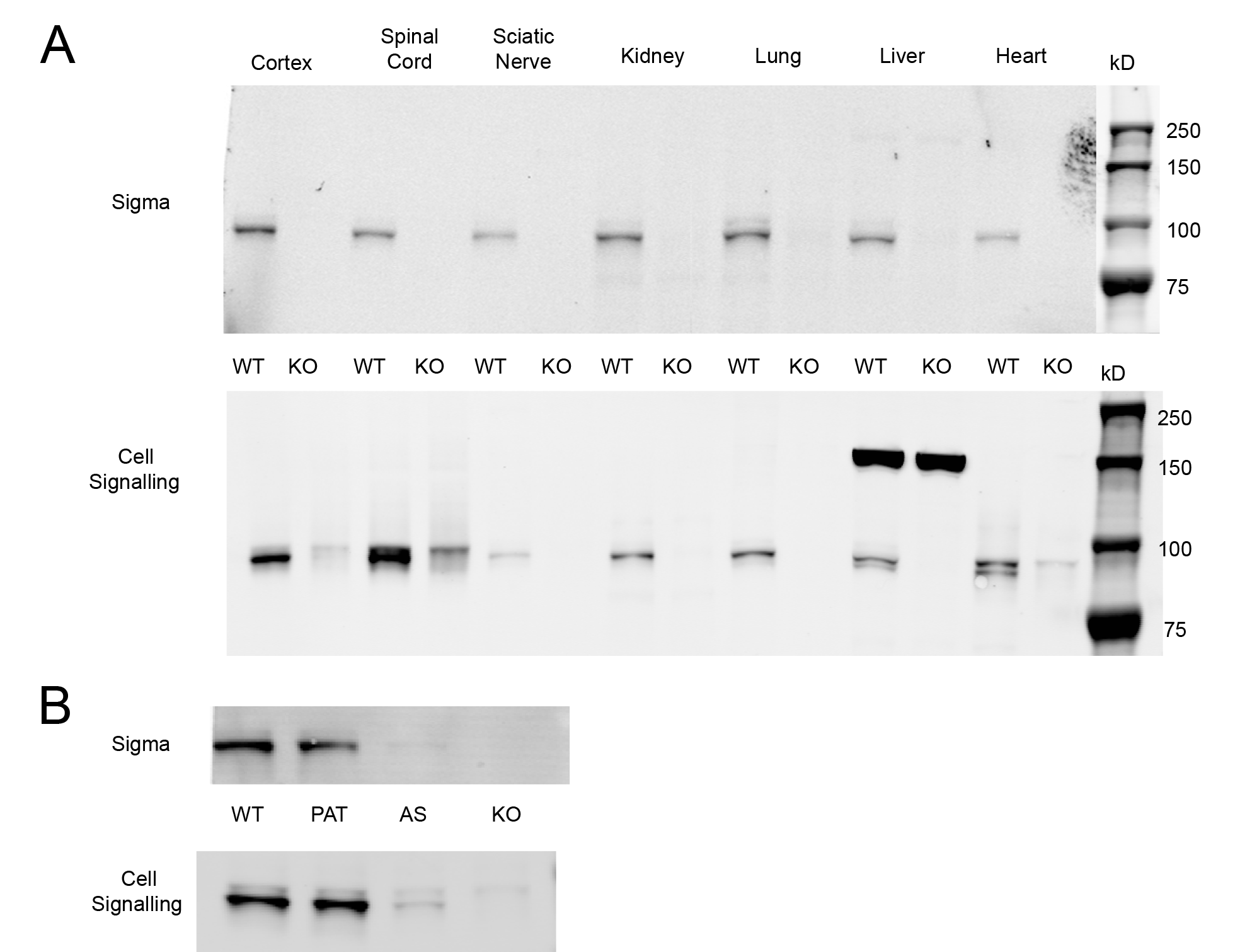

Supplement: S1 Fig — a) Comparison of Ube3a antibodies from Sigma (raised in Mouse) and Cell Signaling (raised in Rabbit) in a panel of tissues known to express Ube3a. WT and KO tissue from tissue was assayed. Top panel shows specificity of Sigma Ube3a with no immunoreactivity at 100 kD in KO tissue in any tissue. Bottom panel shows lack of specificity for Cell Signaling Ube3a with nonspecific bands present at around 100 kD in lysates from cortex, spinal cord, and heart. b) Comparison of antibodies run against WT, paternal deficient Ube3a, AS and KO tissue. As expected, both antibodies have the expected result of WT and paternal deficient sample being nearly indistinguishable, significantly reduced expression in AS tissue and no expression in KO tissue. As noted, a faint non-specific band is recognized by the Cell Signaling antibody. (TIF) [file pone.0124649.s001.tif]
